# Supplementary material for: Inhibitory Effects of Menadione on Helicobacter pylori Growth and Helicobacter pylori-Induced Inflammation via NF-κB Inhibition
Source: Int J Mol Sci. 2019 Mar 7;20(5):1169. doi: 10.3390/ijms20051169 (PMC6429389; doi:10.3390/ijms20051169)
Supplement: Supplementary file 1 [file ijms-20-01169-s001.pdf]

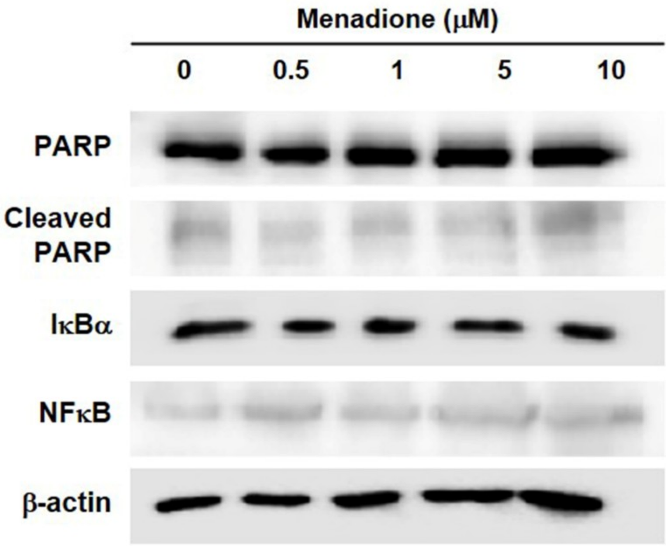

Supplementary figure 1. Western blotting of full-length PARP, cleaved PARP, IκBα, and NF-κB in AGS cells treated with menadione. AGS cells were treated with indicated concentrations of menadione for 12 h. After incubation, cell lysates were collected to conduct Western blotting to detect full-length PARP, cleaved PARP, IκBα, and NF-κB. β-actin was used as an internal control.
